# A Novel, Multi-Institutional Geriatrics Assessment Identifies Knowledge Gaps in Health Professions Trainees

**DOI:** 10.1093/geroni/igaf122.3690

**Published:** 2025-12-31

**Authors:** Apoorva Rangan, Malvika Varma, Rachel Jantea, Mengru Wang, Yochai Shavit, Stephen Pelletier, Andrea Schwartz, Deborah Kado

**Affiliations:** Stanford University, Los Altos Hills, California, United States; Beth Israel Deaconess Medical Center, Boston, Massachusetts, United States; UTHealth Houston, Houston, Texas, United States; University of Washington, Seattle, Washington, United States; Stanford University, Stanford, California, United States; Harvard Medical School, Boston, Massachusetts, United States; VA Boston/Harvard Medical School, Boston, Massachusetts, United States; Stanford School of Medicine, Palo Alto, California, United States

## Abstract

Medical students are expected to care for older people shortly after graduation, yet there are no standardized assessments of geriatrics knowledge. We describe the development and multi-site pilot of the Age-Friendly Knowledge Assessment Tool (AF-KAT), a new assessment evaluating Geriatrics knowledge in clinical trainees. The AF-KAT is a 20-item, case-based multiple-choice questionnaire written by Geriatrics experts and reviewed by general internists and professional editorial staff, including several rounds of peer-review for psychometric and content validity. The AF-KAT questions address the Geriatrics 5M’s and were mapped to updated Geriatrics competencies for medical students and residents. In the first at-scale deployment of this tool, we describe findings from the assessment of 275 graduating medical students across four medical schools (University of Washington, UTHealth Houston, Stanford, and Harvard). Participants self-reported their Geriatrics exposure, attitudes towards aging, and perceived sufficiency of training and completed the AF-KAT. Despite high self-reported preparedness, the mean proficiency score was 60%. Only 25% of students reached a “passing” threshold of 70%. Linear modeling revealed that more curricular exposure (β = 0.37*) and greater understanding of age-friendly systems (β = 0.55*) significantly predicted performance. Negative attitudes toward older people correlated with worse performance (r = -0.20***). This case highlights the feasibility and diagnostic value of using the AF-KAT as an individual-level and program-level assessment tool, with potential uses across health professions trainees. Results underscore the need for a universal and competency-based approach to Geriatrics education, to ensure new physicians are equipped to deliver competent, age-friendly care in an aging society.

